# Psychological Distress in Patients With Asbestos‐Related Diseases and Their Families: A Systematic Literature Review

**DOI:** 10.1002/pon.70051

**Published:** 2025-01-07

**Authors:** Isabella Giulia Franzoi, Maria Domenica Sauta, Michela Bonafede, Giulia Francioso, Alessandra De Luca, Francesca Barbagli, Antonella Granieri

**Affiliations:** ^1^ Department of Psychology University of Turin Turin Italy; ^2^ Epidemiology and Hygiene Department Occupational and Environmental Medicine Italian Workers' Compensation Authority (INAIL) Rome Italy

**Keywords:** asbestos, cancer, caregiver, distress, mental health, mesothelioma, oncology, quality of life

## Abstract

**Background:**

Exposure to asbestos in the workplace is currently recognized as one of the leading causes of work‐related deaths, with more than half of deaths attributable to cancer.

**Aims:**

The aim of this systematic literature review was to investigate the mental health and psychological distress of patients affected by asbestos‐related diseases and their caregivers.

**Methods:**

The review was conducted using the Preferred Reporting Items for Systematic Reviews and Meta‐Analyses. The studies were identified in October 2023 by searching four electronic databases: Scopus, Web of Science, PubMed and PsycInfo/PsycArtcicles. Risk of bias was assessed using the JBI checklist.

**Results:**

Fourteen articles were identified. The studies focused exclusively on the psychological distress of patients with malignant mesothelioma (MM) and their caregivers. MM appears to have traumatic effects on both patients and caregivers, who may experience anxiety and depression, an impoverished emotional life, somatization, social withdrawal, and a deterioration in their quality of life. In addition, a need for information about MM, its progression and associated care tasks was identified, and patients and caregivers reported frequently seeking information from online sources.

**Conclusions:**

Our review has shown that there are still few studies addressing psychological distress in MM patients and their caregivers, and none addressing distress in the context of other asbestos‐related diseases. The somatopsychic consequences of MM in patients and caregivers should encourage institutions and health professionals to develop assessment and intervention models that are tailored to the specific suffering and needs of MM patients and their caregivers and promote their residual vitality.

## 1 Introduction

Furuya and colleagues [1] emphasized the large scale of the phenomenon of asbestos‐related diseases and pointed out that an estimated 2,55,000 deaths per year are attributable to asbestos exposure, of which 2,33,000 are due to occupational exposure. Indeed, exposure to asbestos in the workplace is still considered one of the leading causes of work‐related deaths, with more than half of deaths due to cancer [2]. In countries where asbestos is still manufactured and used, the problem continues as ever: due to the latency period of malignant mesothelioma (MM), which is between 20 and 45 years after exposure, the number of diagnoses is expected to peak in the coming years [3].

It is known that occupational and environmental exposure to asbestos can lead to the onset of various diseases, including asbestosis, pleural plaques, oncological diseases (e.g. lung and laryngeal cancer) and MM [4]. The latter has received particular attention in the literature as it is a rare cancer, accounting for only 0.17% of estimated cancer cases [5], but its only recognized cause appears to be asbestos exposure and its incidence is therefore particularly high in asbestos‐contaminated areas [6, 7]. Furthermore, it has a long latency period between exposure and diagnosis and a poor prognosis: the median survival time is estimated at 4–13 months for untreated patients and 6–18 months for those treated with palliative chemotherapy [8].

Therefore, the psychological literature has also focused particularly on the traumatic consequences of an MM diagnosis, which can lead to the loss of vital aspects of the Self^1^ [9]. Indeed, a cancer diagnosis—regardless of the type of cancer—can affect the somatopsychic balance^2^ of patients and their families, leading to affective dysregulation, difficulties in symbolizing and mentalizing their experiences, an impoverished emotional life, somatization, social withdrawal, and deep feelings of helplessness and vulnerability [10]. However, in the case of MM, the unfavorable prognosis, reduced effectiveness of treatments, occupational etiology, advanced age at diagnosis, and poor quality of life in the later stages of the disease exacerbate the psychological impact and make the experiences of those affected and their family members even more difficult [8, 11, 12, 13]. Reduced quality of life, anxiety, depression, fear and mistrust, somatic and psychosomatic disorders^3^ and post‐traumatic symptoms are common [8, 14]. As for caregivers, the experience of caring for an ill family member can lead to intense physical, psychological, and interpersonal distress [15], which can be even greater for illnesses with a threatening prognosis.

However, the experiences of caregivers are often overlooked, and little space is given to the vulnerabilities of the family unit affected by an oncological disease. At the same time, the information, practice and relational needs of patients and caregivers are often inadequately addressed, and they can feel abandoned in the face of diagnosis, even when they are innovatively treated and supported from an oncology perspective. Furthermore, the fact that MM is a disease that deserves its own attention does not mean that there is no need to examine the experiences of those suffering from other asbestos‐related diseases. Indeed, from a clinical perspective, it is necessary to explore the experiences of patients with different asbestos‐related diseases and their families in order to understand how the different diseases affect their mental health and to develop and provide clinical psychological and psychotherapeutic interventions tailored to their own distinct needs.

This paper therefore presents the results of a systematic literature review aimed at exploring the psychological distress and mental health of patients with asbestos‐related diseases and their families.

## 2 Methods

This systematic literature review was conducted following the Preferred Reporting Items for Systematic Reviews and Meta‐Analyses (PRISMA) 2020 guidelines [16].

### 2.1 Search Strategy

Studies were identified by searching the following electronic databases: Scopus, Web of Science, PubMed and PsycInfo/PsycArtcicles. We used a combination of the following keywords: (mesothelioma OR asbestos) AND (psych* OR mental* OR depress* OR anxi* OR alexithymi* OR distress OR stress OR emoti* OR bereave* OR grief OR pain OR “fear of contagion” OR “perceived risk” OR coping OR “defense mechanism*” OR dissociation OR burden OR fatigue OR “social isolation” OR lonel* OR relationship* OR “quality of life” OR “somatic impact” OR “somatopsychic impact” OR “somato‐psychic impact” OR somatization OR trauma*) AND (patient* OR inpatient* OR outpatient* OR caregiv* OR caretak* OR carer* OR famil* OR spous* OR partner* OR relative* OR parent* OR offspring* OR sibling*).

We have limited our search to the human population and English‐language articles. The articles were retrieved over a 3‐week period until October 25, 2023.

### 2.2 Selection Criteria

The inclusion criteria were as follows:

1. Original studies addressing psychological distress in patients affected by asbestos‐related diseases and their caregivers.
2. Quantitative studies.
3. Journal articles written in English.

The exclusion criteria were as follows:

1. No original studies.
2. Single cases.
3. Qualitative studies.
4. Guidelines.
5. Articles containing data on a population that includes our population of interest, without the ability to extrapolate specific data for our sample of interest.
6. Articles that focus on psychological interventions.

Articles that included samples from different cancer populations were only included if they reported data for MM patients separately.

### 2.3 Screening

**FIGURE 1:**
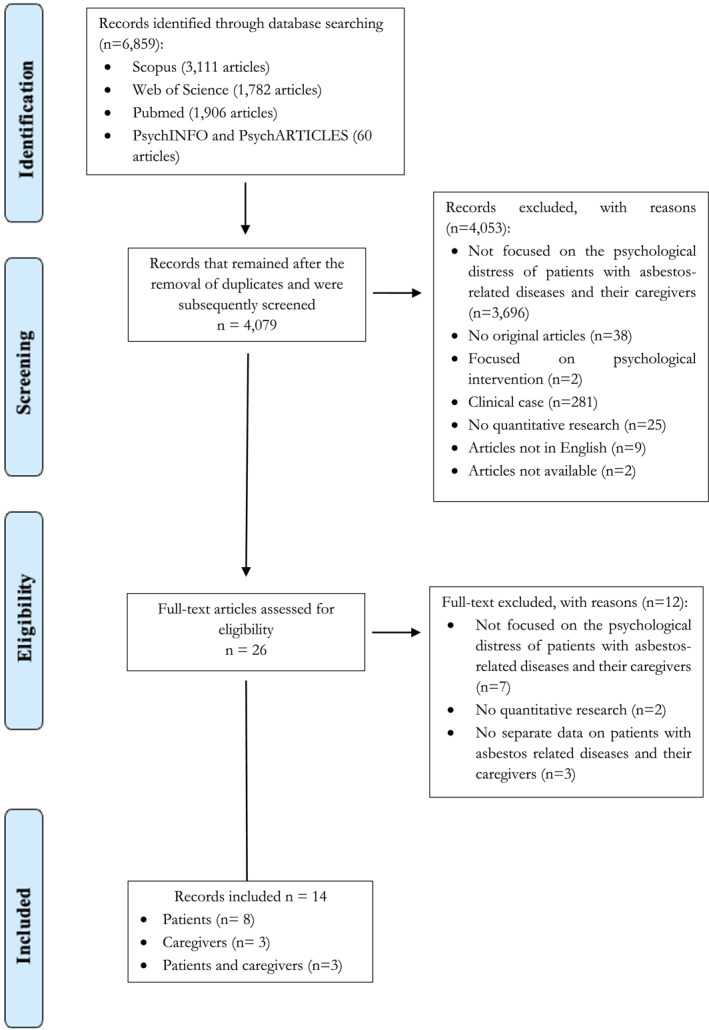
PRISMA flow diagram.

Progressive exclusion was performed by two authors [FB, MDS] who read the title, abstract and full text. In case of disagreement, a third author [IGF] was consulted. Disagreements about the inclusion/exclusion of articles were discussed among the three researchers until consensus was reached. A list of excluded studies was maintained, including the level and reasons for exclusion. The main reasons for exclusion were: Articles that did not focus on the psychological distress of patients with asbestos‐related diseases and their caregivers; no original articles; papers that focused on psychological interventions; clinical cases; no quantitative research; papers without separate data on patients with asbestos‐related diseases and their caregivers; articles not in English; papers not available. The entire process is illustrated in the PRISMA flow diagram (Figure 1).

### 2.4 Quality Assessment

The quality assessment of selected papers was conducted using the JBI Critical Appraisal Checklist for Case Control, Cohort and Cross‐Sectional Studies [17]. Three authors of this paper [ADL, GF, MDS] independently assessed the quality of each study. In case of disagreement, a fourth author [IGF] was consulted. The JBI provides a checklist of items for each type of study that should be assessed for risk of bias. For each item, the researcher is asked to indicate whether or not it is present in the article. The more items on the checklist that are present in an article, the lower the risk of bias. In particular, articles in which more than 70% of the items are present are defined as low risk of bias. Studies with a moderate risk of bias are defined as those in which 50%–69% of the items are present, and studies with a high risk of bias are defined as those in which less than 49% of the items are present. All decisions regarding the scoring system and cut‐off points were approved by all reviewers before the critical appraisal process began.

### 2.5 Data Extraction and Analysis

Data analysis was performed using a standardized data extraction form and included (a) general study details (e.g., authors, title, publication source, year of publication); (b) study type; (c) sample characteristics (e.g., age, sex, country, patients vs. caregivers); (d) measures; and (e) results.

We also checked whether it was possible to perform a meta‐analysis. However, due to the inhomogeneity of the variables measured and the instruments used, a systematic review was preferred to avoid losing data on such different variables. To make the narrative analysis of the selected articles clearer, we decided to organize the results into groups that capture the most important aspects of patients' and caregivers' experiences.

## 3 Results

### 3.1 Study Selection and Risk of Bias Assessment

The electronic database search found 6859 articles. After duplicates had been removed, 4079 articles remained. Of these, 4053 articles were excluded on the basis of title and abstract and 12 articles on the basis of full‐text evaluation (Figure 1). The remaining 14 articles were subject to data extraction and quality assessment for inclusion in this review. Tables 1, 2, 3 summarize the information on the studies.

**TABLE 1:** Results of the Systematic review on psychological distress in patients with asbestos related disease.

ID	Authors	Country	Study	Sample	Measuring tools	Results	Year
2	Nowak AK, stockler MR, Byrne MJ.	Australia	Cross‐sectional study	53 MPM patients	• European Organisation for Research and Treatment of Cancer Quality of Life Questionnaire C‐30 (QLQ‐C30)	• Role function: *M* = 57	2004
					European Organisation for Research and Treatment of Cancer Quality of Life questionnaire (QLQ‐LC13)		
						• Social function: *M* = 67	
						• Emotional function: *M* = 76	
						• Physical function: *M* = 90	
				• Females: 45 (85%) median age: 63 years (range 47–76 aa)		• Cognitive function: *M* = 84	
						• Global health status: *M* = 55	
						• Fatigue, insomnia, dyspnea (QLQC30, and pain (QLQ‐C30 mean scores ranging: 37–42.	
						Cough, dyspnea (LC13, appetite loss, and constipation mean scores ranging: 21–33.	
5	Kao SC, Vardy J, Harvie R, chatfield M, van Zandwijk N, clarke S, Pavlakis N.	Australia	Cross‐sectional study	• *n* = 34 arm A: thalidomide + chemotherapy	Lung cancer symptom scale (LCSS)	• The baseline QoL measures were not significantly different according to the treatment received (*p* ≥ 0.05).	2013
				• *n* = 29 arm B: Thalidomide alone			
				• Females: 7 versus 4			
				• Median age (range): 61 (44–76) versus 67 (50–78)			
				Histology			
				• Epithelial: 14 versus 14			
				• Sarcomatoid: 3 versus 1 mixed/undifferentiated: 17 versus 14			
						Systemic symptoms such as anorexia and fatigue, the summation of overall symptomatic distress, interference with normal activity and global QoL, and the total LCSS score all showed a correlation with blood neutrophil‐to‐lymphocyte ratio (NLR), C‐reactive proteins (CRP) and vascular endothelial growth factor (VEGF) levels (spearman correlation >0.25, *p* < 0.05).	
6	Ribi K, Bernhard J, schuller JC, Weder W, Bodis S, Jörger M, Betticher D, schmid RA, stupp R, Ris HB, stahel RA; swiss Group for clinical cancer Research SAKK.	Switzerland	Cohort study	61 MPM patients	• Rotterdam symptom checklist (RSCL)	Baseline	2008
					Schedule for the evaluation of individual quality of life (SEIQoL)		
				• Females: 4			
				• Median age: 59 years (range 44–72)			
				Histology			
				• Epithelial: 42			
				• Sarcomatoid: 3			
				• Mixed: 14			
						• RSCL QoL: M = 93.0; S.D. = 11.7	
						• RSCL physical symptom distress: *M* = 88.5; S.D. = 8.0	
						• RSCL psychological distress: *M* = 76.8; S.D. = 14.7	
						• SEIQoL: *M* = 70.9; S.D. = 18.9	
						Changes in QoL during treatment	
						• 1 month after surgery: RSCL QoL: *M* = −66; SEIQoL: *M* = −14.	
						• Three months after surgery: RSCL QoL: *M* = −16; SEIQoL scores improved to baseline‐level	
						• 6 months after surgery: RSCL QoL = −15; SEIQoL: *M* = −16	
						• 1 month after surgery: RSCL physical symptom distress: *M* = −14	
						Three months after surgery: RSCL physical symptom distress: *M* = −12	
7	Guglielmucci, F; Bonafede, M; Azzolina, D; Marinaccio, A; Franzoi, IG; Migliore, E; Mensi, C; chellini, E; Romeo, E; Grosso, F; Granieri, A	Italy	Observational multicentric study	149 MM patients	Mesothelioma psychological distress tool‐patients (MPDT‐P)	The first factor “trauma‐related reactions” (13 items) covers a plethora of negative cognitive (e.g., intrusive thoughts and nightmares), emotional (e.g., depressive conditions of hopelessness and loss of interest, death anxieties, and shame) and bodily reactions (e.g., sweating, tachycardia, nausea, and diarrhea).	2022
				• Females: 62 (46%) median age: 71 years		The second factor “claim for justice” (7 items) reflects a reactive/reparative position characterized by feelings of anger and betrayal for having been exposed to a harmful pollutant, along with the desire to obtain economic compensation for it.	
10	Demirjian, CC; saracino, RM; Napolitano, S; schofield, E; Walsh, LE; key, RG; Holland, J	United States of America	Cross‐sectional study	67 MPM patients	• Functional assessment of cancer therapy—Lung (FACT‐L)	Total; Not Depressed; Depressed; Effect size; *p*‐value	
					• Zung self‐Rating depression scale	Brief COPE	
					• Coping Orientation to problems experienced inventory–Brief version (Brief COPE)	• Denial: 1.42 ± 0.60; 1.45 ± 0.72; 1.41 ± 0.58; 0.07; 0.827	
					Social support questionnaire—Short form	• Positive: 2.51 ± 0.86; 3.32 ± 0.75; 2.35 ± 0.79; 1.24; < 0.001	
						• Self‐distraction: 2.45 ± 0.88; 2.86 ± 0.95; 2.36 ± 0.85; 0.58; 0.085	
						• Active coping: 3.16 ± 0.81; 3.32 ± 0.64; 3.13 ± 0.84; 0.23; 0.494	
						• Substance use: 1.07 ± 0.23; 1.05 ± 0.15; 1.08 ± 0.25; −0.15; 0.655	
						• Emotional support: 3.49 ± 0.67; 3.41 ± 0 58; 3.50 ± 0.69; −0.14; 0.683	
						• Instrumental support: 2.81 ± 0.85; 3.05 ± 0.99; 2.77 ± 0.83; 0.33; 0.327	
						• Disengagement: 1.22 ± 0.49; 1.18 ± 0.46; 1.22 ± 0.49; −0.08; 0.799	
						• Venting: 1.89 ± 0.77; 2.20 ± 0.82; 1.84 ± 0.75; 0.48; 0.170	
						• Planning: 2.79 ± 0.93; 2.86 ± 0 98; 2.78 ± 0.92; 0.09; 0.779	
						• Humor: 1.75 ± 0.82; 2 36 ± 1.00; 1.63 ± 0.73; 0.95; 0.005	
						• Acceptance: 3.35 ± 0.70; 3.77 ± 0.47; 3.27 ± 0.71; 0.75; 0.027	
				• Females: 20 (30%) age: 65.61 ± 9.71 (range: 35–83)		• Religiosity: 2.87 ± 1.20; 3.09 ± 1.09; 2.83 ± 1.22; 0.22; 0.509	
						• Self‐blame: 1.28 ± 0.53; 1.23 ± 0.61; 1.29 ± 0.52; −0.11; 0.742	
						FACT‐L	
						• Physical well‐being: 18.60 ± 6.23; 19.27 ± 8.25; 18.47 ± 5.84; 0.13; 0.700	
						• Social/family well‐being: 23.12 ± 3.97; 22.44 ± 4.64; 23.25 ± 3.86; −0.20 0.539	
						• Emotional well‐being: 17.51 ± 4.64; 19.45 ± 3.96; 17.13 ± 4 70; 0.51; 0.130	
						• Functional well‐being: 16 49 ± 5.78; 18 45 ± 7.78; 16 10 ± 5.30; 0.41; 0.220	
						• Lung cancer subscale: 18.63 ± 4.68; 20.18 ± 5.25; 18.32 ± 4.55; 0.40; 0.231	
						• Trial outcome index: 53.72 ± 14.27; 57.91 ± 19.68; 52.90 ± 13.03; 0.35; 0.290	
						• FACT‐G total: 75.73 ± 16.00; 79.62 ± 20.51; 74.96 ± 15.07; 0.29; 0.381	
						• FACT‐L total: 94.35 ± 19.42; 99.80 ± 25.33; 93.28 ± 18.13; 0.34; 0.312	
						Social support questionnaire, short form	
						• SSQ support: 3.87 ± 2.21; 4.44 ± 2 38; 3.75 ± 2.17; 0.31; 0349	
						SSO satisfaction: 5.65 ± 0.56; 5.65 ± 0.49; 5.65 ± 0.58; 0.01; 0.974	
12	Nagamatsu Y, oze I, Aoe K, Hotta K, Kato K, Nakagawa J, Hara K, Kishimoto T, Fujimoto N.	Japan	Cross‐sectional study	133 MPM patients	• EORTC‐QLQ‐C30	EORTC QLQ C‐30	2018
				• Females: 22 (16.5%)			
				• Mean age: 69.3 mean duration of disease (months): 31.0 ± 43.6			
						• Global QOL: *M* = 47.9; SD = 24.9	
						• Physical functioning: *M* = 64.4; SD = 25.8	
						• Role functioning: *M* = 54.1; SD = 30.3	
						• Emotional functioning: M = 70.1; SD = 24.8	
						• Cognitive functioning: *M* = 64.5; SD = 25.7	
						• Social functioning: *M* = 67.0; SD = 28	
						• Fatigue: *M* = 50.8; SD = 26.4	
						• Nausea & vomiting: *M* = 12.9; SD = 21.7	
						• Pain: *M* = 34.7; SD = 29.0	
						• Dyspnea: *M* = 50.1; SD = 29.0	
						• Insomnia: *M* = 36.1; SD = 30.9	
						• Appetite loss: *M* = 38.3; SD = 3 4.7	
					Short version of the core domains of the comprehensive quality of life outcome questionnaire (CoQoLo)	• Constipation: *M* = 38.1; SD = 34.6	
						• Diarrhea: *M* = 14.8; SD = 23.0	
						• Financial difficulties: *M* = 33.1; SD = 31.9	
						Short CoQoLo	
						• Total score: *M* = 48.9; SD = 9.7	
						• Being free from physical pain: *M* = 3.8; SD = 1.9	
						• Being able to stay at one's favorite place: *M* = 5.3; SD = 1.4	
						• Having some pleasure in daily life: *M* = 4.4; SD = 1.7	
						• Trusting physician; *M* = 5.8; SD = 1.5	
						• Feeling like the cause of trouble for others; *M* = 4.0: SD = 1.8	
						• Spending enough time with one's family: *M* = 5.0; SD = 1.6	
						• Being dependent in daily activities: *M* = 5.4; SD = 1.6	
						• Living in calm circumstances: *M* = 5.4; SD = 1.4	
						• Being valued as a person: *M* = 5.4 SD = 1.3	
						• Feeling that one's life was complete, *M* = 4.4 SD = 1.7	
13	Gonzalez‐Ling, A; Vazquez, OG; Bello, ME; Robles, R; Rascon‐Gasca, ML; Lara‐Mejia, L; Heredia, D; Arrieta, O	Mexico	Cross‐sectional study	204 lung cancer patients	• Hospital anxiety and depression scale (HADS)	Anxiety more frequent in females than males: 4.94 versus 2.07, *z* = −4.644, *p* < 0.001	2023
					• Distress thermometer (DT)		
					• EORTC QLQ‐C30		
					EORTC‐LC13		
				• Females: 162 (79%) median age: 61 years (range 24–84)			
						Depression more frequent in females than males: 5.15 versus 2.43, *z* = −3.481, *p* < 0.001	
						Anxiety higher in a second‐line or further lines of treatment than a first‐line treatment (4.89 vs. 3.87, *z* = −2.079, *p* = 0.03)	
						QoL	
						• Cough (70%)	
						• Fatigue (65%)	
						• Loss of appetite (63%)	
						• Pain (62%)	
						• Dyspnea (61%)	
						• Nausea or vomit (51%)	
						• Peripheral neuropathy (48%)	
						• Alopecia (47%)	
						• Financial difficulties 71%	
						• Reduced emotional functioning 54%	
						• Impaired role functioning 45%	
						Patients without psychological disorders VS patients with a psychological disorder	
						• Depression (63.41 ± 23.79 vs. 44.97 ± 24.88; *p* < 0.001)	
						• Distress (63.33 ± 24.42 vs. 51.15 ± 25.38; *p* < 0.001)	
						• Anxiety (63.4 ± 24.2 vs. 47.06 ± 24.72; *p* < 0.001)	
						Spearman correlation test	
						• Positive and moderate association between anxiety, depression (rs = 0.664, *p* = < 0.01)	
						Emotional distress (rs = 0.527, *p* = < 0.01)	
						• Higher levels of anxiety had a negative impact on emotional functioning (rs = −0.527, p = < 0.01) and global QoL (rs = −0.325, *p* = < 0.01), and correlates positively with higher levels of pain (rs = 0.308, *p* = < 0.01), fatigue (rs = 0.300, *p* = < 0.01), and insomnia (rs = 0.353, *p* = < 0.01) depression were associated with lower scores on global QoL (rs = −0.431, *p* = < 0.01), physical (rs = −0.413, *p* = < 0.01), role (rs = −0.404, *p* = < 0.01), emotional (rs = −0.433, *p* = < 0.01), and social functioning (rs = −0.338, *p* = < 0.01), and higher levels of pain (rs = 0.327, *p* = < 0.01), fatigue (rs = 0.367, *p* = < 0.01), and insomnia (rs = 0.332, *p* = < 0.01)	
14	Dooley, JJ; Wilson, JP; Anderson, VA	Australia	Cross‐sectional study	49 MM patients	• Trauma symptom inventory (TSI)	TSI	2010
					• Impact of events scale–Revised (IES‐R)		
					• Center for epidemiologic studies depression Scale (CES‐D)		
					• General health questionnaire (GHQ)		
					Symptom checklist 90–Revised (SCL‐90‐R)		
				• 100% men age: 51.1 ± 6.0			
						• Anxious arousal: 48.63 ± 4.94; 58.15 ± 6.19; 70.10 ± 8.17	
						• Depression: 45.81 ± 3.66; 52.69 ± 6.45; 66.80 ± 10.41	
						• Anger/irritability: 46.75 ± 4.30; 52.23 ± 4.25; 65.40 ± 9.45	
						• Intrusive experiences: 49.69 ± 5.40; 60.08 ± 7.11; 72.60 ± 11.11	
						• Defensive avoidance: 49.56 ± 4.52; 55.23 ± 6.10; 67.90 ± 7.49	
						• Dissociation: 47.44 ± 4.38; 53.92 ± 5.42; 72.50 ± 10.52	
						• Sexual concerns: 48.94 ± 5.09; 53.08 ± 6.29; 62.50 ± 15.55	
						• Dysfunctional sexual behavior: 48.94 ± 7.48; 53.92 ± 10.38; 61.70 ± 15.58	
						• Impaired self‐reference: 45.44 ± 3.20; 51.92 ± 4.11; 69.75 ± 7.36	
						• Tension‐reduction behavior: 46.63 ± 3.00; 52.23 ± 7.69; 66.00 ± 14.64	
						• Overall score: 48.13 ± 3.69; 55.85 ± 5.29; 73.25 ± 8.19	
						IES‐Rb	
						• Avoidancve 1.55 ± 1.04; 2.11 ± 1.10; 2.64 ± 0.91	
						• Re‐experiencing: 1.69 ± 1.01; 2.63 ± 1.21; 3.09 ± 0.93	
						• Hyperarousal: 1.48 ± 1.20; 2.33 ± 1.11; 3.23 ± 0.96	
						SCL‐90‐Ra	
						• Somatisation: 61.31 ± 9.00; 70.31 ± 8.05; 78.25 ± 3.80	
						• Obsessive–compulsive: 60.19 ± 8.38; 67.62 ± 4.77; 80.15 ± 1.76	
						• Interpersonal sensitivity: 52.94 ± 8.47; 65.92 ± 7.39; 77.30 ± 5.31	
						• Depression: 56.13 ± 11.13; 69.00 ± 6.84; 79.75 ± 3.08	
						• Anxiety: 54.75 ± 8.12; 69.23 ± 7.70; 79.75 ± 2.59	
						• Hostility: 51.44 ± 9.88; 61.62 ± 5.17; 70.95 ± 7.95	
						• Phobic anxiety: 53.44 ± 9.22; 65.38 ± 9.08; 76.95 ± 5.80	
						• Paranoid ideation: 53.63 ± 6.30; 59.69 ± 7.47; 73.30 ± 6.41	
						• Psychoticism: 58.00 ± 8.94; 65.77 ± 5.95; 77.80 ± 4.92	
						• Global severity index: 59.00 ± 9.25; 71.31 ± 6.46; 80.75 ± 1.12 distress index: 53.31 ± 6.81; 56.62 ± 6.32; 70.80 ± 6.87	

**TABLE 2:** Results of the Systematic review on psychological distress in caregivers of patients with asbestos related disease.

ID	Authors	Country	Study	Sample	Measuring tools	Results	Year
3	Nagamatsu Y.; sakyo Y.; Barroga E.; Koni R.; Natori Y.; Miyashita M.	Japan	Cross‐sectional study	72 bereaved family members	• Good death inventory (GDI	• GDI mean score: 61.9 ± 15.7	2022
					Short version of the care evaluation scale (CES version 2.0)	CES mean score: 70.3 ± 16.0	
4	Nagamatsu, Y; sakyo, Y; Barroga, E; Koni, R; Natori, Y; Miyashita, M	Japan	Cross‐sectional study	72 bereaved family members	• Patient health Questionnaire‐9 (PHQ‐9)	• 19.4% moderate to severe depression	2022
						• 15.3% suffered of complicated grief (CG)	
						• 56.9% probable CG	
						• 72.2% possible CG	
						• 2.8% both depression and CG.	
						• The PHQ‐9 score was significantly correlated with the BGQ score (*r* = 0.481, *p* (*r* = −0.403, *p* = 0.000), but not correlated with the CES score.	
						Binominal logistic regression model predicting depression	
						• Family financially impacted by patient's MPM: Estimated odds Ratio: 2.569; 95% CI: 1316–5015; *p*‐Value: 0.006	
						• Not compensated by the asbestos‐related health‐damage relief system: Estimated odds Ratio: 7334; 95% CI: 1401–38,374; *p*‐Value: 0.018	
						Binominal logistic regression model predicting CG	
						• Family financially impacted by patient's MPM: Estimated odds Ratio: 3278; 95% CI: 1250–8596; *p*‐Value: 0.016	
						• Not compensated by the asbestos‐related health‐damage relief system: Estimated odds Ratio: 19,210; 95% CI: 1609–229,392; *p*‐Value: 0.020	
						• Received surgery: Estimated odds Ratio: 11,301; 95% CI: 1256–101,649; *p*‐Value: 0.030	
						Not satisfied with the care given when the patient became critical: Estimated odds Ratio: 13,626; 95% CI: 1213–153,009; *p*‐Value: 0.034	
					• Brief grief questionnaire (BGQ)		
					• Good death inventory (GDI)		
					Short version of the care evaluation scale (CES)		
				• Women: 57 (79.2%) age: 62.5 ± 12.2 (range: 32–82)			
8	Bonafede M, chiorri C, Azzolina D, Marinaccio A, Migliore E, Mensi C, chellini E, Romeo E, Grosso F, Franzoi IG, Granieri A, Guglielmucci F.	Italy	Observational multicentric study	144 caregiver of MM patients	Mesothelioma psychological distress tool‐caregivers (MPDT‐C)	The Bayesian exploratory factor analysis revealed an underlying three‐factor structure.	2022
				• Females: 99 (68.75%) age: 56.02 ± 12.87 (range 24–85)			
						The first factor “secondary traumatic stress” (STS) (22 items)	
						STS implies a constellation of reactions characterized as compassion fatigue and post‐traumatic stress symptoms.	
						The second factor “Engagement in caring” (EC) (11 items) contains a wide range of items that reflects different caregiving positions, varying from dysfunctional and detached to more adaptive.	
						The third factor “Meaningful cognitive Restructuring” (MCR) (12 items) reflect attempts made by caregivers to cognitively restructure their traumatic reality, indicating their “need to structure relevant situations in meaningful, integrated ways” and “to understand and make reasonable the experiential world	

**TABLE 3:** Results of the Systematic review on psychological distress in patients with asbestos related disease and their families.

ID	Authors	Country	Study	Sample	Measuring tools	Results	Year
1	Warby A, Dhillon HM, Kao S, Vardy JL.	Australia	Cross‐sectional study	• 78 patients with malignant pleural mesothelioma (MPM)	• Ad hoc surveys on treatments and decision‐making developed from previous interviews with patients, caregivers, and health professionals	Information seeking	2019
				• 106 caregivers			
				Patients:			
				• 15% female			
				• Median age 69 years (range 44–92)			
				• Median time since diagnosis 15 months, long‐term survivors (range 1–82 months)			
				Caregivers:			
				• Median age 68			
				• 91% female			
				Nature of exposure:			
				• 93% occupational			
				• 16% home renovations as adults			
				• 4% childhood exposure			
				1% other			
					Decision Regret scale		
						85% of patients and 86% of caregivers reported seeking information about MPM, mostly online	
						• 36% patients reported it was ‘often’ or ‘almost always’ helpful	
						• 62% of patients and 61% of caregivers reported it was ‘overwhelming’ or ‘somewhat overwhelming’	
						• 67% of patients and 65% of caregivers discussed this information with their treating specialist	
						• 35% of patients and 58% of caregivers would have liked more information	
						Treatment options	
						99% participants reported discussing treatment options with a health professional	
						• Informed about chemotherapy (95% patients and 92% caregivers)	
						• Informed about pleurodesis (78% patients and 77% caregivers)	
						• Informed about radiation therapy (68% patients and 65% caregivers)	
						• Informed about radical surgery (55% patients and 52% caregivers)	
						69% believed that all treatment options were presented	
						Main sources of information	
						• 84% specialist	
						• 54% written information	
						• 37% cancer nurse specialist	
						• 34% general practitioner (GP	
						• 30% nurse‐led education session	
						24% of patients reported declining at least one treatment option offered	
						Involvement of health professionals	
						Patients reported their general practitioner (GP involvement in their care as):	
						• ‘Managing their care’ (10%)	
						• ‘A significant amount’ (26%)	
						• ‘Reasonable amount’ (31%)	
						• ‘Very little’ (23%)	
						• ‘Not at all’ (9%.)	
						Patient and caregiver perceptions of health professionals' communication about their care was that they talked to each other:	
						• ‘Frequently’ (34%)	
						• ‘Almost always’ (15%)	
						• ‘Sometimes’ (21%)	
						• ‘Occasionally’ (11%)	
						• ‘hardly ever’ (14%)	
						Decision‐making about treatment	
						80% of patients felt that they had sufficient information to choose about their treatment.	
						The decision whether or not to have chemotherapy was taken:	
						• Considering the doctor's opinion (24%)	
						• As a joint decision by doctor and patient (18%)	
						• By the doctor (17%)	
						42% patients reported their family had ‘a lot’ or ‘moderate’ influence in their decision regarding chemotherapy	
						83% caregivers were ‘strongly’ or ‘moderately involved’ in the decision‐making process and 75% believed that they and the person they care (d for were in ‘complete agreement’	
						6% patients and 11% caregivers reported speaking to other patient (s about chemotherapy before making a decision: 71% found it helpful	
						7% participants stated that they would have liked to but did not have the opportunity	
						Regrets about whether or not to have chemotherapy	
						• 5% regretted it	
						Complete data for the decision Regret scale were available for 57 patients and 59 caregivers:	
						• Regret was higher in caregivers (*M* = 35.2, SD 28; range 0–95 compared to patients (*M* = 19.9, SD = 19.9; range 0–70; *p* = 0.001	
						Support	
						71% of patients and caregivers reported receiving ‘sufficient’ support	
						Compensation	
						97% of patients and of 65% caregivers sought compensation	
7	Guglielmucci, F; Bonafede, M; Azzolina, D; Marinaccio, A; Franzoi, IG; Migliore, E; Mensi, C; chellini, E; Romeo, E; Grosso, F; Granieri, A	Italy	Observational multicentric study	149 MM patients	Mesothelioma psychological distress tool‐patients (MPDT‐P)	The first factor “trauma‐related reactions” (13 items) covers a plethora of negative cognitive (e.g., intrusive thoughts and nightmares), emotional (e.g., depressive conditions of hopelessness and loss of interest, death anxieties, and shame) and bodily reactions (e.g., sweating, tachycardia, nausea, and diarrhea).	2022
				• Females: 62 (46%) median age: 71 years		The second factor “claim for justice” (7 items) reflects a reactive/reparative position characterized by feelings of anger and betrayal for having been exposed to a harmful pollutant, along with the desire to obtain economic compensation for it.	
9	Bonafede M, Granieri A, Binazzi A, Mensi C, Grosso F, santoro G, Franzoi IG, Marinaccio A, Guglielmucci F.	Italy	Cross‐sectional study	108 patients	• Beck depression Inventory‐II (BDI‐II)	COPE	2020
				• Females: 36 (33.3%)			
				• Age: 66.9 ± 7.4			
				94 caregivers			
				• Females: 68 (72.3%) age: 56.3 ± 14.1.			
					• Davidson trauma scale (DTS)		
					• Coping orientation to problems experienced—new Italian version (COPE)		
					Defense style questionnaire (DSQ‐40)		
						• Social support patients: *M* = 6.5; SD = 14.2	
						caregivers: *M* = 17.7; SD = 15.3	
						• Avoidance strategies patients: *M* = 5.4; SD = 13.5	
						caregivers: *M* = 14.3; SD = 12.3	
						• Positive attitude patients: *M* = 19.7; SD = 16.2	
						caregivers: *M* = 18.1; SD = 14.8	
						• Problem solving patients: *M* = 17.7; SD = 15.0	
						caregivers: *M* = 18.6; SD = 15.6	
						• Turning to religion patients: *M* = 13.7; SD = 11.0	
						caregivers: *M* = 14.5; SD = 11.5	
						DSQ	
						• Sublimation patients: *M* = 5.0; SD = 2.5	
						caregivers: *M* = 5.2; SD = 2.2	
						• Humor patients: *M* = 5.2; SD = 2.6	
						caregivers: *M* = 5.4; SD = 2.2	
						• Anticipation patients: *M* = 5.2; SD = 2.3	
						caregivers: *M* = 5.3; SD = 1.7	
						• Suppression patients: *M* = 5.1; SD = 2.2	
						caregivers: *M* = 4.3; SD = 1.9	
						• Withdrawal patients: *M* = 4.7; SD = 2.3	
						caregivers: *M* = 4.9; SD = 2.0	
						• Pseudo‐altruism patients: *M* = 4.7; SD = 4.4	
						caregivers: *M* = 4.5; SD = 1.8	
						• Idealization patients: *M* = 4.4; SD = 2.3	
						caregivers: *M* = 4.5; SD = 2.3	
						• Reaction formation patients: *M* = 4.6; SD = 2.1	
						caregivers: *M* = 4.9; SD = 2.0	
						• Projection patients: *M* = 3.0; SD = 2.1	
						caregivers: *M* = 3.2; SD = 2.0	
						• Passive aggressive behavior patients: *M* = 3.2; SD = 2.0	
						caregivers: *M* = 3.2; SD = 1.9	
						• Acting out patients: *M* = 4.3; SD = 2.0	
						caregivers: *M* = 4.3; SD = 2.2	
						• Isolation patients: *M* = 3.9; SD = 2.2	
						caregivers: *M* = 3.6; SD = 2.0	
						• Devaluation patients: *M* = 4.5; SD = 2.1	
						caregivers: *M* = 4.3; SD = 2.0	
						• Autistic fantasy patients: *M* = 3.5; SD = 2.6	
						caregivers: *M* = 3.1; SD = 2.3	
						• Denial patients: *M* = 3.3; SD = 2.2	
						caregivers: *M* = 2.7; SD = 1.9	
						• Displacement patients: *M* = 2.8; SD = 1.9	
						caregivers: *M* = 3.1; SD = 1.9	
						• Dissociation patients: *M* = 3.7; SD = 2.4	
						caregivers: *M* = 3.3; SD = 2.0	
						• Splitting patients: *M* = 4.1; SD = 2.0	
						caregivers: *M* = 3.9; SD = 2.2	
						• Rationalization patients: *M* = 5.6; SD = 2.3	
						caregivers: *M* = 5.1; SD = 1.8	
						• Somatization patients: *M* = 2.8; SD = 2.4	
						caregivers: *M* = 2.9; SD = 1.9	
						BDI‐II patients: *M* = 9.7; SD = 8.9	
						caregivers: *M* = 11.4; SD = 0.9	
						DTS	
						• Total score patients: *M* = 20.6; SD = 20.1	
						caregivers: *M* = 31.8; SD = 21.2	
						• Intrusion patients: *M* = 6.0; SD = 7.7	
						caregivers: *M* = 10.1; SD = 8.1	
						• Avoidance/Numbing patients: *M* = 6.5; SD = 7.6	
						caregivers: *M* = 7.7; SD = 6.0	
						• Hyperarousal patients: *M* = 8.7; SD = 9.3	
						caregivers: *M* = 14.1; SD = 11.1	
11	Granieri, A; Tamburello, S; Tamburello, A; casale, S; cont, C; Guglielmucci, F; Innamorati, M	Italy	Cross‐sectional study	27 MPM patients	• The World health organization quality of life assessment (WHOQOL‐BREF	WHOQOL	2013
				• Age: 61.41 ± 8.82			
				40 healthy controls:			
				• Females: 22			
				• Age: 44.63 ± 13.02			
				55 first‐degree caregivers			
				• Females: 43 (78.2%) age: 56.51 ± 13.66			
					Minnesota Multiphasic personality inventory (MMPI 2 ‐RF)	• Physical health patients: 57.28 ± 15.83	
						First‐degree relatives: 63.95 ± 14.54	
						Healthy controls: 75.40 ± 11.58	
						• Psychological health patients: 57.37 ± 15.34	
						First‐degree relatives: 61.09 ± 13.48	
						Healthy controls: 65.83 ± 10.77	
						• Social health patients: 69.17 ± 16.47	
						First‐degree relatives: 65.53 ± 14.97	
						Healthy controls: 62.50 ± 18.14	
						• Environmental health patients: 57.01 ± 16.20	
						First‐degree relatives: 56.23 ± 14.80	
						Healthy controls: 64.53 ± 11.73	
						MMPI 2 ‐RF	
						• RC3 patients: 9.26 ± 3.05	
						First‐degree relatives: 9.25 ± 2.84	
						Healthy controls: 7.33 ± 3.79	
						• MLS patients: 4.74 ± 2.09	
						First‐degree relatives: 2.96 ± 2.01	
						Healthy controls: 2.33 ± 1.62	
						• COG patients: 3.56 ± 2.75	
						First‐degree relatives: 2.29 ± 2.46	
						Healthy controls: 1.60 ± 1.96	
						• HLP patients: 2.59 ± 1.28	
						First‐degree relatives: 2.22 ± 1.26	
						Healthy controls: 1.40 ± 1.87	
						• NFC patients: 4.85 ± 2.43	
						First‐degree relatives: 4.45 ± 2.57	
						Healthy controls: 3.00 ± 2.32	
						• BRF patients: 2.00 ± 1.21	
						First‐degree relatives: 2.67 ± 1.83	
						Healthy controls: 1.45 ± 1.43	

We decided to organize the narrative analysis of the results into five sections:

1. Somatic and psychosomatic symptoms
2. Impairment of quality of life
3. Psychological distress (e.g. anxiety, depression, lack of control, distrust, fear, isolation)
4. Traumatic impact of the diagnosis
5. Information and relational needs

The assessment of the quality of the included articles is presented in Table 4. All 14 articles assessed (100%) were cross‐sectional studies with a moderate risk of bias.

**TABLE 4:** Risk of bias.

	Authors	Item 1	Item 2	Item 3	Item 4	Item 5	Item 6	Item 7	Item 8	Item 9	Item 10	Item 11	Risk of bias
Cross‐sectional study													
1	Warby, A.; Dhillon, H.M.; Kao, S.; Vardy, L.J.	•	•	•	•	•	•	•	•				Moderate
2	Nowak, A.K.; stockler, M.R. & Bryne, M.J.	•	•	•	•	•	•	•	•				Moderate
3	Nagamatsu, Y.; sakyo, Y.; Barroga, E.; Koni, R.; Natori, Y. & Miyashita, M.	•	•	•	•	•	•	•	•				Moderate
4	Nagamatsu, Y; sakyo, Y; Barroga, E; Koni, R; Natori, Y; Miyashita, M	•	•	•	•	•	•	•	•				Moderate
5	Kao SC, Vardy J, Harvie R, chatfield M, van Zandwijk N, clarke S, Pavlakis N	•	•	•	•	•	•	•	•				Moderate
6	Ribi K, Bernhard J, schuller JC, Weder W, Bodis S, Jörger M, Betticher D, schmid RA, stupp R, Ris HB, stahel RA; swiss Group for clinical cancer Research SAKK.Ribi K, Bernhard J, schuller JC, Weder W, Bodis S, Jörger M, Betticher D, schmid RA, stupp R, Ris HB, stahel RA; swiss Group for clinical cancer Research SAKK.	•	•	•	•	•	•	•	•				Moderate
7	Guglielmucci, F; Bonafede, M; Azzolina, D; Marinaccio, A; Franzoi, IG; Migliore, E; Mensi, C; chellini, E; Romeo, E; Grosso, F; Granieri, A	•	•	•	•	•	•	•	•				Moderate
8	Bonafede M, chiorri C, Azzolina D, Marinaccio A, Migliore E, Mensi C, chellini E, Romeo E, Grosso F, Franzoi IG, Granieri A, Guglielmucci F.	•	•	•	•	•	•	•	•				Moderate
9	Bonafede M, Granieri A, Binazzi A, Mensi C, Grosso F, santoro G, Franzoi IG, Marinaccio A, Guglielmucci F.	•	•	•	•	•	•	•	•				Moderate
10	Demirjian, CC; saracino, RM; Napolitano, S; schofield, E; Walsh, LE; key, RG; Holland, J	•	•	•	•	•	•	•	•				Moderate
11	Granieri, A; Tamburello, S; Tamburello, A; casale, S; cont, C; Guglielmucci, F; Innamorati, M	•	•	•	•	•	•	•	•				Moderate
12	Nagamatsu Y, oze I, Aoe K, Hotta K, Kato K, Nakagawa J, Hara K, Kishimoto T, Fujimoto N.	•	•	•	•	•	•	•	•				Moderate
13	Gonzalez‐Ling, A; Vazquez, OG; Bello, ME; Robles, R; Rascon‐Gasca, ML; Lara‐Mejia, L; Heredia, D; Arrieta, O	•	•	•	•	•	•	•	•				Moderate
14	Dooley, JJ; Wilson, JP; Anderson, VA	•	•	•	•	•	•	•	•				Moderate
Color code: Yes = •; No = •; Unclear = •; Not applicable = •													

*Note:* Cross sectional.

JBI Q1.

Were the criteria for inclusion in the sample clearly defined?

JBI Q2.

Were the study subjects and the setting described in detail?

JBI Q3.

Was the exposure measured in a valid and reliable way?

JBI Q4.

Were objective, standard criteria used for measurement of the condition?

JBI Q5.

Were confounding factors identified?

I Q6.

Were strategies to deal with confounding factors stated?

JBI Q7.

Were the outcomes measured in a valid and reliable way?

JBI Q8.

Was appropriate statistical analysis used?

The papers are referenced with the IDs given in Tables 1, 2, 3 to 4.

### 3.2 Overview of Included Studies

All articles address psychological distress in patients with MM and/or their caregivers: we found no studies addressing the psychological impact of other asbestos‐related diseases. Most articles (8; 57.14%) (ID: 2, 5, 6, 7, 10, 12, 13, 14) examined the psychological impact of MM in patients only, 3 (21.4%) (ID: 3, 4, 8) examined it in caregivers only, and 3 (21.4%) (ID 1, 9, 11) examined it in both patients and caregivers. Most studies included both women and men, with the exception of one article (ID 14) in which the sample consisted exclusively of male subjects. Only 4 (28.5%) articles (ID 2, 8, 11, 13) had a majority of female participants in the sample. The age of the patients ranged from 35 to 92 years, the age of the caregivers from 24 to 85 years.

The studies were conducted in the following countries: 4 (28.5%) in Australia (ID 1, 2, 5, 14), 4 (28.5%) in Italy (ID 7, 8, 9, 11), 3 (21.4%) in Japan (ID 3, 4, 12), 1 (7.14%) in Switzerland (ID 6), 1 (7.14%) in the USA (ID 10) and 1 (7.14%) in Mexico (ID 13).

Many studies have investigated the impact of MM by examining the quality of life and general well‐being of affected patients and their caregivers. Data were collected using the following instruments: the European Organization for the Research and Treatment of Cancer (EORTC) Core Quality of Life Questionnaire (QLQ‐C30) [18] (ID 2, 12, 13) and Lung Cancer Module (QLQ‐LC13) [19] (ID 2, 13); the short version of the core domains of the Comprehensive Quality of Life Outcome questionnaire (CoQoLo) [20] (ID 12); a modified version of the Lung Cancer Symptom Scale (LCSS) [21] (ID 5), in which hemoptysis was removed from the scale; the Functional Assessment of Cancer Therapy—Lung (FACT‐L) by Cella and colleagues [22] (ID 10); the World Health Organization Quality of Life–BREF (WHOQOL‐BREF) [23] (ID 11); the Rotterdam Symptom Checklist (RSCL) by de Haes and colleagues [24] (ID 6); the Schedule for the Evaluation of Quality of Life‐Direct Weighting (SEIQoL‐DW) [25]; the General Health Questionnaire (GHQ) [26]; the Symptom Checklist‐90 (SCL‐90) [27].

Quality of care at the end of life was assessed with the short version of the Care Evaluation Scale (CES) [28] (ID 3, 4) and achievement of a good death was measured with the Good Death Inventory (GDI) [29] (ID 3, 4). Complicated Grief (CG) was assessed in one study (ID 4) using the Japanese version of the Brief Grief Questionnaire (BGQ) [30].

Depression was assessed using various diagnostic tests: the Japanese version of the Patient Health Questionnaire‐9 (PHQ‐9) [31] (ID 4); the Beck Depression Inventory, Second Edition (BDI‐II) (ID 9) [32]; the Center for Epidemiologic Studies Depression Scale (CES‐D) (ID 14) [33]; the Zung Self‐Rating Depression Scale (ID 10) by Zung [34].

Anxiety and depression were examined together in a study (ID 13) using the Hospital Anxiety and Depression Scale (HADS) [35].

Post‐traumatic stress symptoms and the burden of living with MM were assessed with the Davidson Trauma Scale (DTS) (ID 9) [36], the Trauma Symptom Inventory (TSI) (ID 14) [37], the Impact of Events Scale–Revised (IES‐R) (ID 14) by Weiss and Marmar [38] and the Mexican version of the Distress Thermometer (DT) [39].

Coping strategies and social support were assessed with the Coping Orientation to Problems Experienced Inventory (Brief COPE) (ID 10) by Carver [40] and its Italian version (ID 9) by Sica and colleagues [41] as well as with the Social Support Questionnaire—Short Form (ID 10) by Sarason and colleagues [42].

To assess the specific profile of psychological distress in this population, including their personality traits and activated defense mechanisms, the Mesothelioma Psychological Distress Tool—Patients (MPDT‐P) (ID 7) by Guglielmucci and colleagues [43], the Mesothelioma Psychological Distress Tool—Caregivers (MPDT‐C) (ID 8) by Bonafede and colleagues [14], the Minnesota Multiphasic Personality Inventory‐2 Restructured Form (MMPI‐2‐RF) (ID 11) by Ben‐Porath and Tellegen [44] and the Defense Style Questionnaire (DSQ‐40) (ID 9) were used [45].

In one study, an ad hoc survey on treatments and decision‐making developed from previous interviews with patients, caregivers, and health professionals (ID 1) was used to assess the experiences of MM patients and their caregivers.

### 3.3 Narrative Analysis

#### 3.3.1 Somatic and Psychosomatic Symptoms

Studies have shown that MM leads to a disruption of somatopsychic balance (ID 2, 6, 11). MM patients had a greater overall feeling of physical debilitation and poorer physical health than their caregivers; they complained more frequently of memory problems and difficulty concentrating (ID 11). During medical treatment (surgery, radiotherapy, chemotherapy and immunotherapy), patients showed an increase in psychological distress and physical symptoms, deterioration in both cognitive and physical function, and worsening of cough, pain, nausea, vomiting, shortness of breath and fatigue (ID 2, 6, 11, 12). MM patients with elevated inflammatory markers, indicating an exaggerated systemic inflammatory response, had an increased symptom burden (ID 5).

#### 3.3.2 Impairment of quality of life

The physical symptoms appear to affect and limit various aspects of quality of life, such as emotional functioning, role behavior, normal activities and overall quality of life (ID 5). In addition, the negative impact of diagnosis and treatment on patients' quality of life affect the survival of MM patients and is associated with increased mortality (ID 5). Active therapy seems to play a critical role: MM patients receiving chemotherapy have similar anxiety and depression scores since diagnosis and 6 months after starting treatment (ID 5). In contrast, patients undergoing surgery show a deterioration in quality of life 1 month after surgery (median change of −66 and −14 for RSCL and SEIQoL, respectively), an improvement 3 months after surgery, and a further deterioration 6 months after surgery (median change: −16) (ID 6).

In patients with mental disorders, global quality of life was worse than in patients without mental impairment: increasing financial problems and physical symptoms combined with lower functioning were also significantly associated with anxiety, depression, and distress (ID 13).

#### 3.3.3 Psychological Distress (e.g. Anxiety, Depression, Lack of Control, Distrust, Fear, Isolation)

Studies suggest that the psychological suffering of MM patients differs from that of other cancers due to several aspects: the limited prognosis and lack of effective treatment options; the burden and fatigue associated with the compensation process; the feelings of guilt and shame that often arise after workplace asbestos exposure; the inability to understand or recognize the various health risks (ID 8, 9).

On an intrapsychic level, anxiety, depression, post‐traumatic symptoms, anger, fear, alienation and somatization may occur (ID, 8, 9, 10, 11). MM patients experience greater emotional suppression compared to their caregivers: this defense mechanism serves to prevent them from thinking and verbalizing the negative emotions associated with cancer, but it actually increases their psychological distress (ID 9).

In addition, patients and caregivers may experience worsening of their family dynamics, social isolation, distrust of institutions, and stigmatization due to death anxieties triggered by MM (ID 3, 4, 6, 8, 9, 10, 11). Caregivers often experience feelings of helplessness/hopelessness and anxiety that restrict their behavior. The sense of uncertainty and loss of control associated with the disease can cause both patients and caregivers great distress and considerable concerns about the progression of a disease that medical treatments often cannot address (ID 3, 4, 6, 8, 9, 10; 11).

To restore their perceived control over a powerless condition and minimize their responsibility for having become ill and exposed their loved ones to an invisible environmental health risk, their aggressive stance may take the form of lawsuits and class actions to obtain economic compensation. However, the legal process to obtain justice and compensation can become a source of stress for patients and caregivers (ID 7,8). Survivors who were not compensated by the compensation system for asbestos‐related health injuries and who felt the financial impact of the patient's MM on the family were more likely to suffer from depression (ID 4).

#### 3.3.4 Traumatic Impact of the Diagnosis

Studies emphasize that the diagnosis of MM can have traumatic impact on patients and caregivers and on the relationship between them. Patients exhibit avoidance strategies that involve cognitive and behavioral efforts to downplay the actual health risks of asbestos exposure (ID 7, 9, 14). Their traumatic stress is associated with health concerns, physical complaints and general psychological impairment (ID 14).

Post‐traumatic symptoms are associated with passive–aggressive behavior and avoidance strategies in MM patients and with acting out in caregivers^4^ (ID 9). The most severe reactions to MM include activation of dysfunctional defense mechanisms and trauma‐related dissociation. Trauma‐related beliefs and emotions that have been repressed from consciousness may intrude into the psyche of MM patients in the form of thoughts or nightmares related to the course of the illness or manifest in physical symptoms and increase the risk of developing post‐traumatic symptoms (ID 9).

#### 3.3.5 Information and Relational Need

A study examining the information and relational needs of patients and caregivers showed that they often seek information about MM after diagnosis, particularly from online sources. The main sources of information are for the 84% specialist, for the 54% written information, for the 37% cancer nurse specialist, for the 34% general practitioner and for the 30% nurse‐led education session (ID 1). Patients rated these as “often” or “almost always” helpful, while caregivers as “overwhelming” or “somewhat overwhelming”. Patients mainly shared the information with their treating specialists or with family members and friends. In addition, patients expressed a desire for more information about the expectations associated with their illness, while caregivers wanted to be informed about what to expect when caring for a MM patient (ID 1). About treatment options 99% of patients and caregivers reported discussing treatment options with a health professional and the 69% believed that all treatment options were presented (ID 1). 24% of patients reported declining at least one treatment option offered (ID 1). 80% of patients felt that they had sufficient information to choose about their treatment. The decision whether or not to have chemotherapy was taken: considering the doctor's opinion (24%), as a joint decision by doctor and patient (18%) or by the doctor (17%) (ID 1). 42% patients reported their family had “a lot” or “moderate” influence in their decision regarding chemotherapy. 83% caregivers were “strongly” or “moderately involved” in the decision‐making process and 75% believed that they and the person they cared for were in “complete agreement” while 6% patients and 11% caregivers reported speaking to other patients about chemotherapy before making a decision: 71% found it helpful (ID 1).

## 4 Discussions

Although our systematic literature review aimed to investigate the psychological distress and mental health of patients with asbestos‐related diseases and their caregivers, we only found articles that focused on patients affected by MM and their family members. Thus, the distress associated with asbestos‐related diseases such as asbestosis, pleural thickening and pleural plaques is still not adequately addressed. Indeed, the scientific and institutional interest in this subject has often favored an epidemiological view aimed at quantifying the impact of asbestos‐related diseases on the physical health of the population, while neglecting their dramatic impact on mental health.

The World Health Organization [46] estimates that 125 million people worldwide are exposed to asbestos in the workplace each year, and the International Labor Organization states that more than 1,07,000 workers die each year from a related disease. In countries such as Brazil, China, Kazakhstan and Russia, where asbestos is still manufactured and exported [47], the issue of asbestos‐related diseases and the plight of affected patients and their families should be on the agenda of treatment and mental health services. However, in our systematic literature review, we did not find any studies conducted in these countries. Thus, there appears to be a lack of tools to address the plight of patients with asbestos‐related diseases and their families precisely where more diagnoses are expected.

The results of this systematic review confirm the impact of MM on the somatopsychic balance of patients and caregivers.

Many studies in this systematic review have investigated the impact of MM by examining the quality of life and general well‐being of affected patients and their caregivers, showing significant impairment in quality of life [11, 43, 48, 49, 50], and a range of somatic and psychosomatic symptoms [50, 51, 52]. A diagnosis of MM can affect the ability of patients and caregivers to regulate their emotions and to symbolize and mentalize the illness experience, altering their sense of personal coherence. When unable to find words to describe their emotional experiences, patients and caregivers may resort to archaic forms of mental functioning^5^ that do not use language and respond to their mental pain on a somatic level [53]. Not being able to assign a personal meaning to what is happening in their own lives makes it difficult to understand oneself and others on an emotional and cognitive level, increasing the risk of relational conflict and isolation [10].

The traumatic impact of the diagnosis on those affected and their families can be very great [11, 43, 48] and lead to depressive symptoms and feelings of hopelessness, helplessness and despair [12, 51]. Post‐traumatic symptomatology [11, 43] can occur not only at the time of diagnosis, but also during treatment and adjustment to later life [54]. Caregivers have been shown to be more traumatized than patients with MM and more likely to report intrusive thoughts of death and physiological hyperactivation. One possible cause is guilt, a key emotion in the experience of cancer caregivers, which may affect their psychosocial and somatic adjustment to cancer [11]. In the case of asbestos exposure, the sense of guilt and shame that often occurs after workplace asbestos exposure, along with the inability to understand or recognize the various health risks, is a feeling that seems to pervade patients and reinforces the traumatic nature of the disease [14].

Our systematic literature review also shows that the mental health of both patients and caregivers can be affected: anxiety, depression, distrust, fear and isolation may occur [8, 11, 12, 13, 14, 49, 50, 51]. In particular, depressive symptoms appear to be an overarching theme in the various studies of patients with MM [48, 55] and they appear to worsen with disease progression [11]. Regarding gender, significant differences between men and women were observed in patients in terms of behavior as well as coping strategies and defense mechanisms [11]. In line with the literature on other cancer patients [12, 56], women were more depressed and experienced more traumatic symptoms than men and used more coping strategies [11]. As the psychological burden of MM differs from that of other cancers due to its etiology and its particular symptomatology and prognosis, two studies focused on the preliminary validation of questionnaires designed to identify the specificity of the illness experience of patients with MM [43] and their caregivers [14].

In relation to the specific distress and mental health of caregivers, it is extremely relevant and alarming that we found only 6 articles addressing the experiences of caregivers [11, 12, 13, 14, 51, 57]. This finding is not surprising, as a lack of studies focusing on caregivers has already been noted in the literature on other types of rare cancers [10]. However, it is alarming as caregivers of patients with rare tumors such as MM are at higher risk of caregiver burden compared to other types of cancer [11, 58]. In addition, their suffering may go unnoticed because they may avoid seeking support for fear of taking time away from their caregiving role. Moreover, the psychological distress associated with the caregiving experience, especially if left untreated, can also have a negative impact on the grieving process [53, 59].

Unsurprisingly, this review found that patients and caregivers need more information about the illness and its treatment [57].

Finally, it is interesting to note that the number of quantitative studies on asbestos‐related diseases is still very low compared to the need for in‐depth studies on this topic. This is not conducive to the development of research intervention protocols aimed at assessing the psychological suffering of patients with asbestos‐related diseases and their families. Instead, it is important to analyze the disease experience in more detail to help clinicians and researchers examine possible differences related to the various occupational and environmental contexts that caused the disease.

We believe that the results of our systematic review allow to point out some important directions for future research. The experiences of patients with asbestos‐related diseases other than MM and their family members should be further investigated. A comparison with other types of occupational or environmental contamination‐related diseases would also be interesting. It would also be important to follow the evolution of the effects of exposure to asbestos in communities where it is still processed and manufactured compared to communities where it has been banned. Finally, it is clear how necessary it is to focus on integrated research projects that always include psychology, to increase the number of quantitative studies and to enrich them with qualitative data.

## 5 Clinical Implications

Pollution related to industrial production is one of the current global crises that is receiving more and more attention on the international agenda.

Although the literature emphasizes that living in asbestos‐contaminated areas, in constant contact with disease and death, can have a strong impact on the somatopsychic balance of the exposed community, leading to the clinical manifestation of important psychopathological aspects, we found few studies that investigated the psychological suffering and mental health of patients with asbestos‐related diseases and their family members. In particular, we only found studies focusing on patients with malignant mesothelioma and their caregivers.

Indeed, scientific interest in this topic is still focused on an epidemiologic perspective aimed at quantifying the impact of asbestos‐related diseases on the physical health of the population, while neglecting the dramatic effects on mental health. Such an imbalance between the attention given to the physical health and mental health of patients and caregivers is no longer sustainable.

Furthermore, we believe that the results of this study are of fundamental benefit to all healthcare professionals working with MM patients and their caregivers. Indeed, mental health professionals need to increase their knowledge of this particular disease and its impact on patients and families and provide interventions tailored to the needs of this special population. In addition, other healthcare professionals should also consider in their clinical practice the unmet needs of patients and family members and the aspects of their intrapsychic and interpersonal functioning that can impact all care relationships, sometimes interfering with the ability to make balanced decisions about one's health and jeopardizing treatment adherence. This would allow, firstly, to calibrate communication between doctor, nurse and patient differently and, secondly, to identify the highest risk cases that should be referred to the clinical psychologist. Interdisciplinarity in this context is essential for effective treatment.

## 6 Limitations

This systematic literature review has several limitations. The results may be affected by the heterogeneity of the instruments used and by the exclusion of qualitative studies, which may have led us to overlook important results. It is also conceivable that many important findings were overlooked, particularly because only English‐language articles were included in this study, seemingly limiting the inclusion of important findings.

## 7 Conclusions

Our systematic review emphasizes that psychological characteristics of patients and caregivers need to be explored and considered in order to direct research and clinical practice towards a more modern planning of health services that can give qualitative and multidisciplinary meaning to quantitative data.

Structuring multidisciplinary interventions that target the actual suffering and needs of patients and caregivers can help them improve their psychophysiological balance^6^ and their ability to regulate and symbolize emotions and mentalize experiences. Care protocols that integrate research and intervention with the aim of providing a rich and detailed description of the complex dynamic nature of the illness experience and the outcomes of interventions in terms of effectiveness, efficiency and processes involved are therefore more necessary and significant than ever to address the suffering of patients with MM and their families [60].

## Data Availability

Data sharing is not applicable to this article as no new data were created or analyzed in this study.

## Endnotes

^1^
In a psychoanalytic perspective, we refer to the Self as the representations about the person herself when she is the object of her own subjective experience. The encounter with the diagnosis of MM can affect the vitality and integration of such representations.

^2^
By somatopsychic balance we mean a state of equilibrium of the mind‐body unit, which is characterized by an integration of the physical and psychological dimensions of experience.

^3^
This means both disorders that directly affect the body and disorders whose symptoms are physical but have no specific organic etiology.

^4^
Defense mechanism consisting of aggressive and violent behavior aimed at the unreflective and uncontrolled realization of unconscious desires and impulses in order to cope with an unresolved emotional conflict.

^5^
That is, modes of functioning that are influenced by unconscious and nonverbal processes, characterized by undifferentiation and the tendency to experience intense emotions in a globalizing and fragmentary way.

^6^
Psychophysiological balance refers to the ability to regulate the reactions of the autonomic nervous system and their emotional correlates.
